# Draft genome sequence of *Bacillus amyloliquefaciens* subsp. *plantarum* strain Fito_F321, an endophyte microorganism from *Vitis vinifera* with biocontrol potential

**DOI:** 10.1186/s40793-018-0327-x

**Published:** 2018-11-01

**Authors:** Cátia Pinto, Susana Sousa, Hugo Froufe, Conceição Egas, Christophe Clément, Florence Fontaine, Ana C Gomes

**Affiliations:** 1 Biocant - Biotechnology Innovation Center, Cantanhede, Portugal; 2 SFR Condorcet - FR CNRS 3417, University of Reims Champagne-Ardenne, Induced Resistance and Plant Bioprotection (RIBP)- EA 4707, BP1039, Cedex 2 51687 Reims, France; 3 Center for Neurosciences and Cell Biology (CNC), Faculty of Medicine, University of Coimbra, Polo I, 1st floor, Rua Larga, 3004-504 Coimbra, Portugal

**Keywords:** Genome sequencing, *Bacillus amyloliquefaciens subsp. plantarum*, Fito_F321 strain, Grapevine-associated microorganism, Biocontrol, Endophytic microorganism

## Abstract

*Bacillus amyloliquefaciens subsp. plantarum* strain Fito_F321 is a naturally occurring strain in vineyard, with the ability to colonise grapevine and which unveils a naturally antagonistic potential against phytopathogens of grapevine, including those responsible for the *Botryosphaeria* dieback, a GTD disease. Herein we report the draft genome sequence of *B. amyloliquefaciens* subsp. *plantarum* Fito_F321, isolated from the leaf of *Vitis vinifera* cv. Merlot at Bairrada appellation (Cantanhede, Portugal). The genome size is 3,856,229 bp, with a GC content of 46.54% that contains 3697 protein-coding genes, 86 tRNA coding genes and 5 rRNA genes. The draft genome of strain Fito_F321 allowed to predict a set of bioactive compounds as bacillaene, difficidin, macrolactin, surfactin and fengycin that due to their antimicrobial activity are hypothesized to be of utmost importance for biocontrol of grapevine diseases.

**Electronic supplementary material:**

The online version of this article (10.1186/s40793-018-0327-x) contains supplementary material, which is available to authorized users.

## Introduction

*Bacillus amyloliquefaciens* is a species from the genus *Bacillus**,* genetically and phenotypically related to *B. subtilis**,* *B. vallismortis*, *B. mojavensis**,* *B. atrophaeus**,* *B. methylotrophicus**,* *B. siamensis**,* *B. velezensis**,* *B. licheniformis**,* and *B. pumilus**,* which altogether form the *B. subtilis* group [1–9]. Taxonomic problems involving the species *B. velezensis**, B. amyloliquefaciens* subsp. *plantarum,* *B. methylotrophicus* and *B. oryzicola* had been recently reported [10]*.* In order to avoid this taxonomic misunderstanding, a more recent study proposed *B. amyloliquefaciens* subsp. *plantarum* as a later heterotypic synonym of *B. velezensis**,* based on phylogenomic analysis [10]*.* Another study also reinforced that *B. amyloliquefaciens**,* *B. velezensis* and *B. siamensis* should be kept as singular species across their clade however, and due to their close relationship, these species should be included in the “operational group *B. amyloliquefaciens**”* within the *B. subtilis* group [11].

*B. amyloliquefaciens* is ubiquitously distributed, Gram-positive, rod-shaped, aerobic and endospore-forming bacteria. Together with other different *Bacillus* species from the *Bacillus subtilis* group, *B. amyloliquefaciens* has been reported to develop beneficial relationships with plants by promoting growth, improving resistance to environmental stress or having important biological activities for plant diseases control [12–14]. These species produce a variety of antimicrobial compounds, such as bacteriocins, antifungal compounds such as lipopeptides, namely iturins and fengycins, and siderophores [15, 16]. Given its biocontrol potential, aligned with its physiological characteristics, such as UV light and heat resistant spores, long shelf life [17] and their advantageous characteristics for formulation, this microorganism is an environmental-friendly alternative to agrochemicals. Indeed, some of *B. amyloliquefaciens* strains are commercially available as biological control agents or generic plant growth promoters [18, 19].

Altogether these characteristics prompted us to explore the *B. amyloliquefaciens* subsp. *plantarum* strain Fito_F321, a naturally occurring strain in vineyards that we have isolated from grapevine leaves in the Bairrada appellation - Portugal. In this study, we have obtained the draft genome sequence of *B. amyloliquefaciens* subsp. *plantarum* strain Fito_F321, analysed it and compared it with known genome sequences of representative related species, to gain knowledge on the genes involved in plant interaction with grapevine, as well as the genes conferring antimicrobial activity, and thus to evaluate the potential of this strain for further viticulture and agronomic applications.

## Organism information

### Classification and features

**Fig. 1:**
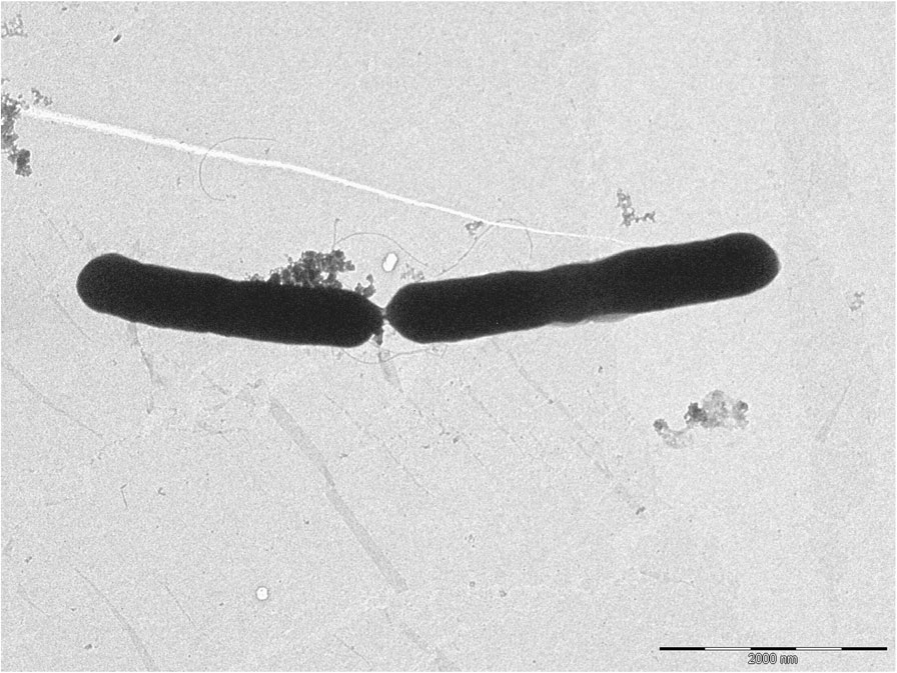
Transmission electron micrograph of Bacillus amyloliquefaciens subsp. plantarum strain Fito_F321. Bar: 2 μm

Strain Fito_F321 was isolated from *Vitis vinifera* cv. Merlot at Bairrada appellation – Cantanhede, Portugal during the 2012 vine cycle. The samples collection was authorized by the private owner, who is fully acknowledged in this paper, and no specific permissions were required for this activity. Briefly, leaf tissues were homogenised in a sterile saline solution (0.85% NaCl) with a sterile pestle. The bacterial isolates were then obtained after plating the homogenised leaves on PDA medium and incubation for 24 h at 28 °C. Sub-cultures were then carried out on the same culture medium until obtaining pure colonies that were further assigned to an isolation code. Microscopic analysis showed that strain Fito_F321 is a Gram-positive and rod shape microorganism (Fig. 1). The classification and general features of strain Fito_F321 are listed in Table 1.

**Table 1:** Classification and general features of Bacillus amyloliquefaciens subsp. *plantarum* strain Fito_F321, according to the MIGS recommendations [69]

MIGS ID	Property	Term	Evidence code^a^
	Classification	Domain *Bacteria*	TAS [70]
		Phylum *Firmicutes*	TAS [71–73]
		Class *Bacilli*	TAS [74, 75]
		Order *Bacillales*	TAS [72, 76]
		Family *Bacillaceae*	TAS [72, 77]
		Genus *Bacillus*	TAS [72, 78]
		Species *Bacillus amyloliquefaciens*	TAS [1, 79]
		Strain: Fito_F321	
	Gram stain	Gram-positive	IDA
	Cell shape	Rod-shaped	IDA
	Motility	Motile	NAS
	Sporulation	Spore-forming	NAS
	Temperature range	unreported	
	Optimum temperature	28 °C	IDA
	pH range; Optimum	6–9, 6.5	IDA
	Carbon source	Organic carbon compounds	NAS
MIGS-6	Habitat	Leaf, grapevine	IDA
MIGS-6.3	Salinity	0–6% (*w*/*v*); salt tolerant	IDA
MIGS-22	Oxygen requirement	Aerobic	NAS
MIGS-15	Biotic relationship	free-living	IDA
MIGS-14	Pathogenicity	Non-pathogen	NAS
MIGS-4	Geographic location	Cantanhede, Portugal	IDA
MIGS-5	Sample collection	2012	IDA
MIGS-4.1	Latitude	40°19′40.11″N	
MIGS-4.2	Longitude	8°32′59.54″O	
MIGS-4.4	Altitude	90 m	

^a^Evidence codes – IDA: Inferred from Direct Assay; TAS: Traceable Author Statement (i.e., a direct report exists in the literature); NAS: Non-traceable Author Statement (i.e., not directly observed for the living, isolated sample, but based on a generally accepted property for the species, or anecdotal evidence). These evidence codes are from the Gene Ontology project [80]

**Fig. 2:**
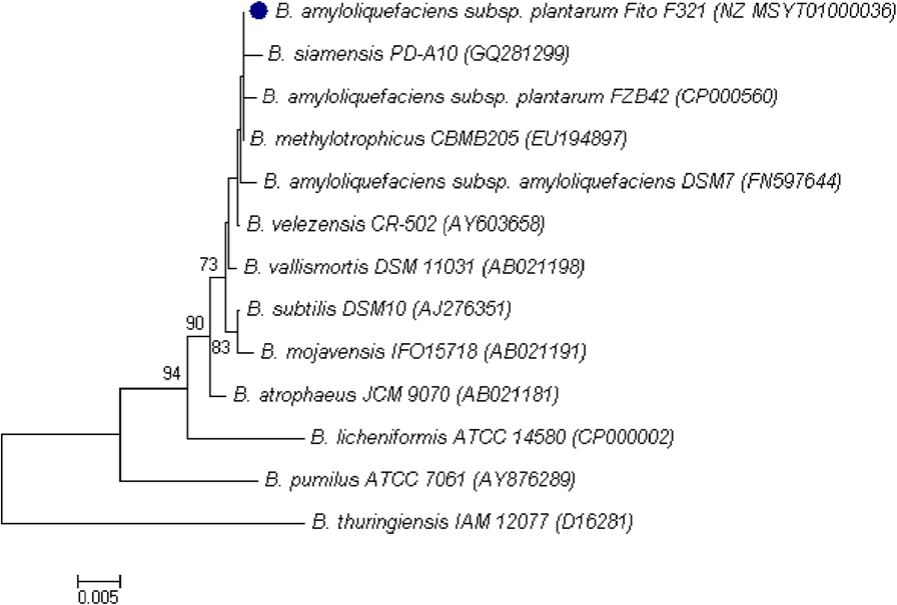
Phylogenetic tree highlighting the taxonomic relation of *B. amyloliquefaciens* subsp. *plantarum* strain Fito_F321() based on 16S rDNA amplicon within the Bacillus clade. Only type strains are included. The GenBank accession numbers are shown in parentheses. Sequences were aligned using ClustalW 1.6 [81]. The phylogenetic tree was constructed by using the Neighbor-Joining method [23] and evolutionary distances were computed by the Maximum Composite Likelihood method [24] within MEGA 7.0 [25]. There were a total of 1380 positions in the final dataset. Numbers at the nodes are bootstrap values calculated from 1000 replicates; only bootstrap values > 70 are indicated

Strain Fito_F321 was taxonomically identified by combining the analysis of the 16S rRNA gene sequence using both SILVA database [20] and EzBioCloud [21], and by genome comparisons. In SILVA the 16S rRNA sequence of strain Fito_F321 showed 99% of similarity to *B. amyloliquefaciens* subsp. *plantarum* AS43.3 (CP003838) and to *Bacillus amyloliquefaciens* subsp. *plantarum* SQR9 (CP006890), both non-type strains. A last updated available, reclassified these two strains as *Bacillus velezensis*. In the other hand, results obtained from EzBioCloud showed a 99.93% similarity of strain Fito_F321 to *B. velezensis* CR-502 (type strain). Given these results, the 16S rRNA gene sequence of strain Fito_F321 and other representative related and type strains species available on GenBank [22] were then selected for phylogenetic analysis (Fig. 2). The phylogenies were generated using the Neighbor-Joining method [23] and evolutionary distances were computed by the Maximum Composite Likelihood method [24] with 1000 bootstrap replicates. Phylogenetic analysis was conducted in MEGA 7.0 [25]. Phylogenetic analysis of the 16S rRNA revealed that strain Fito_F321 is positioned in the same group as *B. amyloliquefaciens* subsp. *plantarum* FZB42, *B. siamensis* PD-A10 and *B. methylotrophicus* CBMB205 and is closest to *B. amyloliquefaciens* subsp. *amyloliquefaciens* DSM7 and *B. velezensis* CR-502. To overcome the difficulties of strain Fito_F321 classification, a comparison of genome sequences between type and other strains of both *B. amyloliquefaciens* and *B. velezensis* species was performed according to the methodology proposed by Chun et al. [26] and is fully presented in the section Comparisons with other genomes. Overall, our results showed that strain Fito_F321 is closer to *B. amyloliquefaciens* subs. *plantarum* FZB42, with a DDH estimate of 85.90% (> 70%) and an ANI similarity of 98.40% (≥95–96%), than to *B. amyloliquefaciens* subsp. *amyloliquefaciens* DSM7 (DDH estimate of 55.30% and ANI similarity of 94.15%). Thus, and according to this data, strain Fito_F321 was classified as a *B. amyloliquefaciens* subsp. *plantarum*.

#### Extended feature descriptions

The physiological and biochemical features of *B. amyloliquefaciens* subsp. *plantarum* strain Fito_F321 were analysed to explore the mechanisms behind its antagonistic potential, namely its ability to produce hydrolytic enzymes, presence of siderophores and phosphate solubilization. The tolerance to pH and salinity conditions were also tested. All tests were performed in triplicate. Given the enzymatic production, the amylase, cellulase, lipase, pectinase, protease and urease activity were screened under in vitro conditions by using specific culture media. Results were expressed as positive activity, when a clear halo around strain colony was observed, and the enzymatic index (EI) was calculated through the ration between the average diameter of the degradation halo (clear zone) and the average diameter of the colony growth. The strain Fito_F321 was able to produce all enzymes under in vitro conditions except ureases. Amongst them, cellulases had the higher enzymatic index (10.50 ± 0.20), followed by pectinases (5.44 ± 0.39). This strain was also able to produce siderophores and to solubilise phosphate under in vitro conditions. Overall, these phenotypic features are of high interest, since they are intimately involved in the biocontrol action. Further, this strain was able to grow between pH 6.0–9.0, with an optimal growth at pH 6.5, and grew under up to 6% NaCl. Interestingly, the morphology of Fito_F321 colonies was altered with salt concentration, and colonies became smaller with increasing NaCl concentration in the culture media. It is recognised that excess of soil salinity reduces both plant growth and yield thus, salt tolerant strains may confer plant tolerance against these abiotic stresses [27].

## Genome sequencing information

### Genome project history

*B. amyloliquefaciens* subsp. *plantarum* strain Fito_F321 was selected for sequencing as a part of an ongoing project that focuses on the deep characterization of the grapevine-associated microorganisms and their natural antagonistic potential. Thus, its specific antagonistic activity against important grapevine pathogens, such as grey mould or grapevine trunk diseases, together with its physiological and biochemical unique features such as the ability to growth on a range of pHs and salinity conditions, the production of siderophores, the phosphate solubilisation and the high enzymatic activity, were the drivers for its sequencing.

Sequencing of the wild-type *B. amyloliquefaciens* subsp. *plantarum* strain Fito_F321 genome was performed at Biocant, Portugal and the draft genome sequencing project has been deposited at DDBJ/ENA/GenBank under the Bioproject PRJNA360208, Biosample ID SAMN06205151 and the accession number MSYT00000000. The version described in this paper is version MSYT01000000. A summary of the project is shown in Table 2.

**Table 2:** Project information

MIGS ID	Property	Term
MIGS 31	Finishing quality	Draft-genome
MIGS-28	Libraries used	Rapid Library Preparation Method GS FLX+ Series XL+
MIGS 29	Sequencing platforms	GS FLX Titanium Sequencing Kit XL+
MIGS 31.2	Fold coverage	41X
MIGS 30	Assemblers	GS Assembler, version 2.9
MIGS 32	Gene calling method	Prodigal, GenePRIMP
	Locus Tag	BVY13
	Genbank ID	MSYT00000000
	Genbank Date of Release	05/01/2018
	GOLD ID	–
	BIOPROJECT	SAMN06205151
MIGS 13	Source Material Identifier	Fito_F321
	Project relevance	Biocontrol, Grapevine, GTD

### Growth conditions and genomic DNA preparation

*B. amyloliquefaciens* subsp. *plantarum* strain Fito_F321 was grown in Luria-Agar medium at 28 °C for 24 h. The genomic DNA was extracted by using the Wizard Genomic DNA Purification kit (Promega, Madison, USA), following the standard protocol for Gram- positive bacteria. The DNA integrity was checked by 0.8% agarose gel electrophoresis, the concentration was determined by using Quant-iT PicoGreen dsDNA Assay Kit (Thermo Fisher Scientific) and quality assessed with NanoDrop spectrophotometer (Thermo Scientific, USA). Prior to genome sequencing, the quality of the isolated DNA and the molecular identity was confirmed by the sequencing of the 16S rRNA gene.

### Genome sequencing and assembly

A DNA library was built from 1 mg of high-quality genomic DNA. Briefly, genomic DNA was fragmented by nebulization and the sequencing adaptors ligated to create double stranded DNA libraries. After quality assessment by using high sensitivity DNA analysis kit (Agilent Technologies) and library titration with KAPA library quantification kit (Kapa Biosystems), the final genome fragments were pyrosequenced in the GS FLX+ system (Roche, 454 Life Sciences), using GS FLX Titanium Sequencing Kit XL+ at Biocant (Cantanhede, Portugal). The sequencing reads were assembled with the GS Assembler, version 2.9 (Roche, 454 Life Sciences) using the default parameters. The sequencing produced 285,879 reads with an average length of 580 bases. The final assembly yielded − 54 contigs, a genome coverage of 41-fold and generated a genome of 3.86 Mb (Table 2).

### Genome annotation

The structural and functional annotations were performed using the Prokaryotic Genome Prediction pipeline [28]. Prediction of non-coding RNA genes and miscellaneous features were performed with the PGP pipeline by using tRNAscan-SE [29], RNAMMer [30] and PILERCR [31]. Coding sequences were predicted with Prodigal [32] and automatically corrected by PGP pipeline based on the GenePRIMP algorithm [33]. Functional annotation of protein coding genes was carried out under Prokaryotic Genome Prediction pipeline in InterProScan [34] against Pfam database [35], TIGRFAM [36], Hamap [37], PIRSF [38], PRINTS [39], SMART [40], SUPERFAMILY [41], ProSite [42] databases and RPS-BLAST against Clusters of Orthologous Groups (COG) database [43]. The product name of the identified coding sequences (CDSs) was assigned by using Pfam database, TIGRFAM and COG annotation [44]. The CDSs that were not assigned to a specific product with these databases were named as hypothetical proteins.

## Genome properties

The genome statistics are provided in Table 3, and genome visualisation was performed on Artemis version 16.0.0 [45]. The draft genome sequencing of *B. amyloliquefaciens* subsp. *plantarum* strain Fito_F321 was distributed across 54 contigs with an estimated genome size of 3,856,229 bp and an average of GC content of 46.53%. The genome analysis showed that Fito_F321 strain’ genome contained 3657 protein coding genes predicted, 95 RNAs and without any CRISP elements. The predicted protein encoding genes showed a total length of 3,424,790 bp which represents 88.81% of the total genome size. Of these, 2697 proteins were assigned to a COG functional category across 20 categories (Table 4). The majority of protein-coding genes were assigned as *function unknown* (264 proteins) and *general function prediction only* (306 proteins), which all together represents 15.59% of the protein encoding genes (Table 4). The proteins not assigned in COGs (960 proteins) represent 26.25% and the *amino acid transport* (269 proteins), *transcription* (227 proteins) and *carbohydrate transport and metabolism* (191 proteins) were the followed categories with 7.36%, 6.21% and 5.22%, respectively. Interestingly, the *defense mechanisms* included 43 protein-coding genes, which represent about 1% of the annotated genome, and included β-lactamase (class C), multi-drug efflux pumps as ATP-binding cassette (ABC) transport and the multidrug and toxic compound extrusion (matE), antimicrobial peptides (AMPs) and lanthionine synthetase component C-like protein (LANCL).

**Table 3:** Genome statistics

Attribute	Value	% of Total^a^
Genome size (bp)	3,856,229	100
DNA coding (bp)	3,424,790	88.81
DNA G + C (bp)	1,794,204	46.53
DNA scaffolds	54	–
Total genes	3846	100
Protein coding genes	3657	95.09
RNA genes	95	2.47
Pseudo genes	94	2.44
Genes in internal clusters	NA	–
Genes with function prediction	2790	72.54
Genes assigned to COGs	2697	70.12
Genes with Pfam domains	3241	84.27
Genes with signal peptides	2,48	6.45
Genes with transmembrane helices	2500	65.00
CRISPR repeats	0	0.00

^a^The total is based on either the size of genome in base pairs or the total number of genes in the predicted genome

**Table 4:** Number of genes associated with general COG functional categories

Code	Value	%age^a^	Description
J	158	4.32	Translation, ribosomal structure and biogenesis
A	0	0.00	RNA processing and modification
K	227	6.21	Transcription
L	97	2.65	Replication, recombination and repair
B	1	0.03	Chromatin structure and dynamics
D	34	0.93	Cell cycle control, Cell division, chromosome partitioning
V	43	1.18	Defense mechanisms
T	105	2.87	Signal transduction mechanisms
M	136	3.72	Cell wall/membrane biogenesis
N	41	1.12	Cell motility
U	40	1.09	Intracellular trafficking and secretion
O	78	2.13	Posttranslational modification, protein turnover, chaperones
C	156	4.27	Energy production and conversion
G	191	5.22	Carbohydrate transport and metabolism
E	269	7.36	Amino acid transport and metabolism
F	78	2.13	Nucleotide transport and metabolism
H	122	3.34	Coenzyme transport and metabolism
I	117	3.20	Lipid transport and metabolism
P	149	4.07	Inorganic ion transport and metabolism
Q	85	2.32	Secondary metabolites biosynthesis, transport and catabolism
R	306	8.37	General function prediction only
S	264	7.22	Function unknown
–	960	26.25	Not in COGs

^a^The total is based on the total number of protein coding genes in the genome

## Insights from the genome sequence

A total of 111 metabolic pathways were identified using the KEGG annotation and included, several metabolism pathways (as alanine, aspartate and glutamate, fructose, mannose, galactose, glutathione, methane, nitrogen, pyruvate, sulphur, tryptophan or starch and sucrose), glycolysis, TCA cycle, fatty acid biosynthesis, glucosinolate biosynthesis, antibiotic biosynthesis (neomycin, kanamycin, gentamicin, puromycin, streptomycin or tetracycline) or degradation pathways of noxious compounds (atrazine, benzoate, bisphenol, dioxin, ethylbenzene, limonene, pinene, naphthalene, polycyclic aromatic hydrocarbon or toluene). In general, and as previously described, the metabolic pathways identified showed that the majority of protein-coding genes are involved in the amino acid metabolism (7.36%), carbohydrate metabolism (5.22%), energy metabolism (4.27%) and lipid metabolism (3.20%).

### Plant-bacteria interactions

The genome of *B. amyloliquefaciens* subsp. *plantarum* strain Fito_F321 was also analysed for genes contributing directly or indirectly for plant-growth promotion (PGP) and biocontrol activities (Additional file 1: Table S1):

#### Colonisation, adhesion, and movement of bacteria across plant root

It is recognized that a crucial feature of a successful plant growth promoter microorganism, as well as of a biocontrol agent relies on its competence for plant colonisation, notably at roots level [46]. Overall a colonisation process may involve a plant surface adhesion/attachment and a bacterial biofilm formation [47]. The *B. amyloliquefaciens* *subsp. plantarum* strain Fito_F321 genome encodes a set of proteins involved in flagella biosynthesis, such as *fliZ* (BVY13_00370), *flgC* (BVY13_14075), *flhF* (BVY13_00340), *flhA* (BVY13_00345), *flhB* (BVY13_00350), *fliR* (BVY13_00355), *fliQ* (BVY13_00360), *fliP* (BVY13_00365) or chemotaxis, namely *cheA* (BVY13_00325), *cheD* (BVY13_00310), *cheV* (BVY13_01575) and *che*W (BVY13_00320). *B. amyloliquefaciens* subsp. *plantarum* strain Fito_F321 also displays a swarming motility, which allows a rapid surface colonization [48]. Herein, genes encoding for *swrA* (BVY13_02415), *swrB* (BVY13_00300) and *swrC* (BVY13_18860) were predicted. Overall, the swarming motility requires both flagella biosynthesis and surfactant production [48]. Other genes such as hook-associated proteins - *flgK* (BVY13_02315), *fliD* (BVY13_02345), or *hag flagellin* (BVY13_02340) can be expressed in response to root exudates secreted by plant roots. Bacterial flagellins can interact with host and are involved in elicitation of general plant immune response [49]. Furthermore, this strain may also produce biofilms. Indeed, the sporulation transcription factor *spo0A* (BVY13_05345) was here identified, and has an important role on biofilm formation, by repressing the expression of AbrB [50, 51]. *Spo0A* is essential for surface-adhered cells prior to transition to a three-dimensional biofilm structure [50, 51].

#### Plant-growth promotion

*B. amyloliquefaciens* subsp. *plantarum* strain Fito_F321 encodes proteins that enhance the plant growth such as those involved in the biosynthesis of indole-3-acetic acid, a plant auxin. Herein, genes encoding for tryptophan, the main precursor of IAA [52], were identified and include *trp* genes such as *trpA* (BVY13_06245), *trpB* (BVY13_06240) or *trpE* (BVY13_06220). Going forward, the synthesis of volatile compounds, as 2,3-butanediol and acetoin, released by some *Bacillus* strains, may also enhance the plant growth promotion and be involved in the eliciting induce systemic resistance [53]. Herein, a set of genes that catalyse the 2,3-butanediol pathway, such as butanediol dehydrogenase *bdhA* gene (BVY13_17360), acetolactate synthase *als* and *alsD* (BVY13_14285, BVY13_09195) and acetolactate decarboxylase *alsD* (BVY13_14290) were identified. Regarding nitrogen fixation, several *nif* genes were not identified among Fito_F321 genome, though other genes involved in nitrate reduction pathways were predicted. Further, a scaffold protein *nifU* (BVY13_03720) and a cysteine desulfurase *nifS,* which are involved in the Fe-S cluster assembly and required for the activation of nitrogenase, where identified. Another feature of *B. amyloliquefaciens* subsp. *plantarum* strain Fito_F321 is the *nirK* gene responsible for the nitric oxide synthase, a signalling molecule that protects Gram-positive strains from antibiotics and oxidative stress [54, 55]. Regarding the phosphate solubilisation, no *pqq* genes were predicted for this bacterial strain. These genes encode a pyrroloquinoline quinine, a PGP agent involved in the phosphate solubilisation process [56]. However, Fito_F321 strain displayed a phytase activity (BVY13_15080) that contributes to the subsequent use of phosphorous by the plant. This activity is important for the plant growth under phosphate limitation [57, 58]. These predictions are in agreement with the in vitro results obtained by using the Pikovskaya culture medium, which unveiled the ability of Fito_F321 strain to solubilise phosphate. Another important feature is that this strain encodes an inositol 2-dehydrogenase (BVY13_11530), important for the inositol catabolism. Inositol or other inositol derivatives are end-products of phytate degradation, abundant in the plant rhizosphere and can be use by microorganisms as carbon sources [57, 59].

An indirect PGP effect can also be mediated through the siderophores production. Siderophores are iron (Fe)- specific chelating small molecules secreted by bacteria and have high affinity with ferric ionic from soils and surrounding environments [60], thus increasing the bioavailability of Fe for plants, by promoting its solubilisation. On other hand, the siderophores production by BCAs may also confer a clear competition for the available carbon sources, allowing for plant colonisation in detriment with other microorganisms. The *B. amyloliquefaciens* *subsp. plantarum* strain Fito_F321 encodes genes for ABC transporters for iron and iron uptake, which was further supported by the genome analysis using antiSMASH 3.0 [61] that also predicted siderophores.

#### Biocontrol activity

*B. amyloliquefaciens* subsp. *plantarum* strain Fito_F321 revealed high potential to produce bioactive secondary metabolites (2.32%) with important biocontrol activities. In agreement with the genome analysis using antiSMASH 3.0 [61], 13 secondary metabolites gene clusters were identified (Additional file 2: Table S2). Amongst them, *B. amyloliquefaciens* subsp. *plantarum* strain Fito_F321 encoded 4 polyketide synthases clusters, 4 nonribosomal peptides synthases clusters and 1 hybrid PKS-NRPS clusters. Thus, 3 types of antibacterial polyene PKs can be produced, comprising bacillaene, difficidin, macrolactin and butirosin; 2 types of lipopeptides as fengycin, bacilysin, surfactin; as well as siderophore bacillibactin. In addition, the remaining 4 clusters were predicted to produce secondary metabolites including ladderane, lantipeptide or terpene cyclase, namely a putative squalene-hopene cyclase (Additional file 2: Table S2).

#### Antimicrobial resistance

In the meantime, the strain Fito_F321 encodes antimicrobial resistance genes (Additional file 1: Table S1) such as bacitracin (bcr - BVY13_11500), fosfomycin (*fosB* - BVY13_12675) and tetracycline (BVY13_08560) [62]. Regarding bacitracin and fosfomycin resistance genes, these are antibiotics that interfere with peptidoglycan synthesis of the bacterial cell wall [63, 64]. Given the bacitracin, herein multiple genes encoding for ABC transporter system were identified, which are associated with bacitracin resistance. Tetracycline antibiotics inhibit the bacterial ribosome, and thus, protein synthesis [65]. In Fito_F321 strain genome, the resistance to tetracycline occurs via active efflux (BVY13_08560).

### Comparisons with other genomes

To further characterize the extent of which *B. amyloliquefaciens* subsp. *plantarum* strain Fito_F321 differentiates from other strains, genome comparisons of strain Fito_F321 were carried out with the genomes of four types trains, namely *B. amyloliquefaciens* subsp. *plantarum* FZB42, *B. amyloliquefaciens* subsp. *amyloliquefaciens* DSM7, *B. velezensis* KCTC 13012 and *B. velezensis* NRRL B-41580, and other 23 complete genomes of non- type strains of *B. amyloliquefaciens,* including related species that show ≥98.7% 16S sequence similarity. For this, both GGDC 2.1 web server [66], using the DSMZ phylogenomics pipeline [67] to estimate the DNA-DNA hybridization, and the JSpecies WS web server [68] to estimate the Average Nucleotide Identity through pairwise comparisons of genomes were applied. The estimate DDH was calculated with the formula two at the GGDC website, which is the recommended for draft genomes and the ANI values were calculated using the MUMmer software (ANIm) as described by Richter and Roselló-Móra (2009) [68]. This analysis allowed for the calculation of the intergenomic distances between genomes and the probability of belonging to the same species. The general comparison is shown in Table 5 and the intergenomic distances, through the DDH estimate and ANI, are in Table 6. Given the analysis with type-strains, results have shown that *B. amyloliquefaciens* subsp. *plantarum* strain Fito_F321 had a lower distance with *B. amyloliquefaciens* subs. *Plantarum* FZB42 with a DDH estimate of 85.90% and a probability to correspond to the same species of 94.14%. These results were also supported by the ANI analysis where both strains reached a similarity of 98.40%, with 95.22% of the genome aligned. Contrary, *B. amyloliquefaciens* subsp. *amyloliquefaciens* DSM7 was the strain most distant from strain Fito_F321, with a DDH estimate of 55.30% and a probability to correspond to the same species of 35.90%. The same comparative results were performed for non-type strains. Herein, *B. amyloliquefaciens* subsp. *plantarum* SQR9 showed the lower intergenomic distance and the higher similarity with *B. amyloliquefaciens* subsp. *plantarum* strain Fito_F321.

**Table 5:** Comparative analysis of the genome features of *B. amyloliquefaciens* subsp. *plantarum* strain Fito_F321 with both *B. amyloliquefaciens* and *B. velezensis*. Details for each genome was completed according to the information available at NCBI and EzBioCloud

	Strain	GB acession number	Isolation source	Country	Genome size (Mb)	G+C content (%)	Protein-coding sequences	tRNA coding genes	rRNA
	*B. amyloliquefaciens* subsp. *plantarum* strain Fito_F321	MSYT00000000	Leaves (vineyard)	Portugal	3.86	46.54%	3.697	86	5
Type-strains	*B. amyloliquefaciens* subsp. p*lantarum* FZB42	CP000560	Soil (sugar beet)	Germany	3.92	46.50%	3.687	89	31
	*B. amyloliquefaciens* subsp. a*myloliquefaciens* DSM 7	FN597644	Not available/unknown	Germany	3.98	46.10%	3.870	94	30
	*Bacillus velezensis* KCTC 13012	LHCC00000000.1	River velez	Spain	4.04	46.30%	3.806	80	9
	*Bacillus velezensis* NRRL B-41580	LLZC00000000.1	River Velez	Spain	4.03	46.30%	3.790	80	9
Non type-strains	*B. amyloliquefaciens* subsp. p*lantarum* SQR9	CP006890	Cucumber rhizosphere	China	4.12	46.10%	3.902	72	21
	*Bacillus amyloliquefaciens* WS-8	CP018200.1	Soil	China	3.93	46.50%	3.670	86	27
	*Bacillus amyloliquefaciens* CC178	CP006845.1	Cucumber phyllosphere	South Korea	3.92	46.50%	3.702	86	27
	*Bacillus amyloliquefaciens* KHG19	CP007242.1	Fermented soybean paste	South Korea	3.95	46.60%	3.698	89	31
	*Bacillus amyloliquefaciens* Y2	CP003332.1	Wheat rhizosphere	China	4.24	45.90%	4.038	87	31
	*B. amyloliquefaciens* subsp. *plantarum* AS43.3	CP003838	Surface of a wheat spike	USA	3.96	46.60%	3.669	89	31
	*Bacillus amyloliquefaciens* UMAF6614	CP006960.1	Not available/unknown	Not available/unknown	4.01	46.50%	3.754	83	27
	*Bacillus amyloliquefaciens* B15	CP014783.1	Grape skin	China	4.01	46.50%	3.759	90	31
	*Bacillus amyloliquefaciens* UMAF6639	CP006058.1	Not available/unknown	Not available/unknown	4.03	46.30%	3.741	83	27
	*Bacillus amyloliquefaciens* S499	CP014700.1	Soil	Democratic Republic of the Congo	3.93	46.60%	3.720	81	24
	*Bacillus amyloliquefaciens* IT-45	CP004065.1	Not available/unknown	Not available/unknown	3.93	46.60%	3.726	95	30
	*Bacillus amyloliquefaciens* LFB112	CP006952.1	Chinese herbs	China	3.94	46.70%	3.684	94	32
	*Bacillus amyloliquefaciens* Y14	CP017953.1	Rhizosphere of peanut	China	3.96	46.40%	3.741	87	27
	*Bacillus amyloliquefaciens* LM2303	CP018152.1	Wild yak dung	China	3.99	46.70%	3.771	86	27
	*Bacillus amyloliquefaciens* L-S60	CP011278.1	Soil	China	3.90	46.70%	3.662	91	28
	*Bacillus amyloliquefaciens* L-H15	CP010556.1	Cucumber seedlings	China	3.91	46.70%	3.666	84	28
	*Bacillus amyloliquefaciens* MBE1283	CP013727.1	Korean traditional alcoholic beverage	South Korea	3.97	46.50%	3.725	86	27
	*Bacillus amyloliquefaciens* RD7-7	CP016913.1	Fermented soybean paste	South Korea	3.69	46.30%	3.501	87	27
	*Bacillus amyloliquefaciens* MT45	CP011252.1	Daqu	China	3.90	46.10%	3.752	81	24
	*Bacillus amyloliquefaciens* SRCM101267	CP021505.1	Food	South Korea	4.07	45.90%	4.014	87	27
	*Bacillus amyloliquefaciens* LL3	CP002634.1	Fermented food	South Korea	4.00	45.69%	3.935	72	22
	*Bacillus amyloliquefaciens* TA208	CP002627.1	Soil	China	3.94	45.80%	3.891	70	19
	*Bacillus amyloliquefaciens* XH7	CP002927.1	Not available/unknown	Not available/unknown	3.94	45.80%	3.889	75	22

**Table 6:**
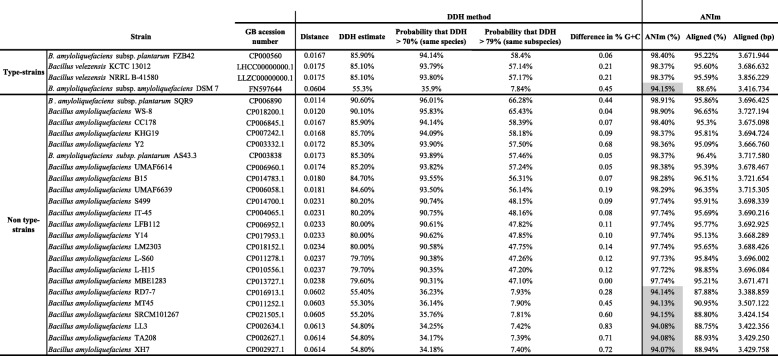
Comparative analysis of the *in-silico* genome distances between *B. amyloliquefaciens* subsp. *plantarum* strain Fito_F321 withboth *B. amyloliquefaciens* and *B. velezensis*, through the DNA-DNA hybridization (DDH method) and average nucleotide identities (ANI)

The nucleotide sequences were download from GenBank and the respective accession numbers are shown in the table. *In-silico* DNA-DNA hybridization (DDH) was calculated by using the Genome-to-Genome Distance Calculator (GGDC 2.1) [67] and ANI values were computed through pairwise genome comparison by using the MUMmer software [68]. Values with grey colour are of a below cut-off (< 95%)

## Conclusions

In this study, we have characterized the genome of *B. amyloliquefaciens* subsp. *plantarum* strain Fito_F321, a natural grapevine-associated microorganism, which was isolated from grapevine leaves. Given its genomic and physiological characteristics, this microorganism may provide an interesting model to study the plant-microbial interactions and their role in grapevine protection. The intergenomic distances amongst genomes showed that *B. amyloliquefaciens* subsp. *plantarum* strain Fito_F321 is highly close to type strain *B. amyloliquefaciens* subs. *plantarum* FZB42, with a DDH estimate value of 85.90% and a ANIm value of 94.14%, and more distant to the type strain *B. amyloliquefaciens* *subsp. amyloliquefaciens DSM7.*

The predicted gene compounds of *B. amyloliquefaciens* subsp. *plantarum* strain Fito_F321 such as bacillaene, difficidin, macrolactin, surfactin, fengycin and siderophore, together with other protein-coding genes herein presented, are of utmost importance for its biocontrol activities and could explain its positive plant-microbial interactions, as well as its role on the natural protection of vineyard. Thus, these gene clusters suggest that the strain Fito_F321 can produce bioactive compounds of biocontrol value, which represents a source of novel bioactive compounds and that may be essential for the grapevine protection in the pursue of a more sustainable viticulture.

## Additional files

Additional file 1: **Table S1.** General overview of genes involved in bacterium-plant interaction in *B. amyloliquefaciens* subsp. *plantarum* strain Fito_F321. (XLSX 11 kb)

Additional file 2: **Table S2.** Secondary metabolite gene clusters identified. (XLSX 14 kb)

## Abbreviations

BCA: Biological control agent
GH: Glycoside hydrolase
GTD: Grapevine Trunk Diseases
PGP: Plant growth promoting
